# How do we make indoor environments and healthcare settings healthier?

**DOI:** 10.1111/1751-7915.12430

**Published:** 2016-10-17

**Authors:** Jack A. Gilbert

**Affiliations:** ^1^ The Microbiome CenterDepartment of SurgeryUniversity of ChicagoChicagoIL60637USA; ^2^ The Division of BioscienceArgonne National LaboratoryLemontIL60439USA; ^3^ The Marine Biological LaboratoryWoods HoleMA02543USA

## Abstract

It is now well accepted that our modern lifestyle has certain implications for our health (Schaub *et al*., 2006), mainly as a result of our willingness to remove ourselves from the biological diversity of our natural environments (Roduit *et al*., 2016), while still being drawn inextricably to interact with it (Kellert and Wilson, 1995). Much of our interaction with the biological world is shaped by our interaction with the microbiological world. The bacteria, fungi, viruses, archaea and protists that comprise the microbiome of this planet, are also key to the development and normal functioning of our bodies. Our immune system is built to shepherd our microbial exposure, ensuring that microbial organisms that we need are kept close (but not too close), and that less‐desirable organisms are expelled or killed before they can do too much damage. By moving from a life interacting with nature on a regular basis, to a life in which we isolate ourselves physically from natural microbial exposure, we may have instigated one of the great plagues of the 21st century; chronic immune disorders.

The buildings in which we live and work may be inadvertently detrimental to our chronic health. There is an obvious trade off here. In environments with epidemic disease, it is essential that we remove ourselves from waste streams that can propagate the infective agents. As people have always liked to gather into ever‐larger villages, towns and cities, it has become necessary to build infrastructure that delivers clean water and food, and takes away waste products. In the absence of these services, and especially if people are ignorant of the problems caused by poor sanitation, disease will spread as people dispose of waste and use available water without taking appropriate precautions to reduce negative health consequences. Over the last few hundred years, especially since the mid‐19th century, the public and government came to realize that disease was being spread by dangerous microorganisms, and therefore that more public works were needed to eradicate them. Over the last 100 years that common sense has transmuted into an out‐and‐out war on microbes, with most people's motto, especially in the healthcare industry, being ‘The only good microbe, is a dead microbe.’

This ideological approach to ‘hygiene’ filtered down into everyone's lives, and has been exemplified by an advertising industry keen to sell products that would facilitate the public's germophobia. This has reached a peak, whereby it is now possible to buy microbe‐killing cleaning agents, surface materials that kill bacteria on site, and even air‐filters that are designed to ensure sterility. There is a surprising lack of data to suggest that any of these precautions have significant health benefit when employed in countries that have adequate health care, vaccination rates and public works that provide clean water, food and removal of sewage. There is, however, a large body of evidence growing that overt cleanliness may be detrimental to our health. With our clean, bleached homes, antimicrobials, and processed air and where even our water is chlorinated and food pre‐wrapped and sterilized, it is no shock that our immune systems, which have been expecting continuous bombardment, are now over‐reacting.

One place we should expect cleanliness and hygiene is our hospitals. People with communicable disease or poorly functioning immune systems need to be kept in an environment where microbes are held at bay. But even here there is need to consider ways to stimulate the immune system to help people heal. Despite expectation, many infections that occur after surgery may be due to bacteria or viruses that were already present in the patient. To prevent these infections, we may not need antibiotics, instead we may want to consider ways to manage the microbiome to prevent it from expressing a virulent lifestyle. In addition, when a patient is healing post treatment, it is possible that controlled microbial exposure may actually help to promote recovery (Defazio *et al*., 2014). So even in hospitals maybe, we should be considering how to manage microbial exposure, rather than attempting to eliminate it entirely.

The purpose of this perspective was not to review the evidence, but to postulate potential solutions that may require a research renaissance to develop. One research anecdote on which we have been working recently comes from studying the microbial exposure of agrarian peoples, and the impact of disrupted exposure on the development of asthma. We have demonstrated previously that Amish families whose children grow up on farms, interacting with animals and the farming environment on a regular basis, have a substantially lower rate of asthma compared with the Hutterites who live a similar technology‐free life, but whose children are not allowed on the farm (Stein *et al*., 2016). Currently, we are proposing intervention studies whereby we expose Hutterite children to farm animals and observe whether this helps to protect against asthma. The hypothesis is that bacteria, viruses and fungi associated with the animals help to train the immune system, as well as helping to build a microbiome in the gut that protects children against asthma development.

Similar interventions could be put in place for other children, for example, those in urban settings who develop atopy, including food allergies, eczema, etc. Through our studies of bacteria, fungi and viruses in the built environment, and through efforts to compare the microbiome of homes between families with atopy and those without (Fujimura *et al*., 2014), it will be possible to identify microorganisms that may protect against atopy onset. But, how would exposure best be mediated. We know that animal exposure can be beneficial; even dogs can provide protection (Fall *et al*., 2015), with evidence that this is mediated through microbial exposure (Fujimura *et al*., 2014). So the simplest way may be to just expose children to more animals. However, in our modern world this is not always possible. Children growing up in urban environments are unlikely to have ready access to animals, and unless there is a shift in how urban environments are managed it is unlikely this will be an effective means of exposure. One way that we have been investigating is to impregnate building materials with microorganisms; essentially to create living walls, ceilings and floors, even carpet materials. Many bacteria can survive in a quiescent state, or as spores, waiting for the right conditions to germinate. Germination or rapid growth usually occurs following the introduction of water, but it can also happen if a person acquires the microbe so it can flourish in their warm, moist body.

How could microbially active materials actually work? First, microbial cells or particles in the case of viruses, can be dried down and added as a coated layer to most materials, or alternatively can be integrated at some stage of material development so that the material is suffused with these agents. If we can design materials with a three‐dimensional structure, to include pores and spaces that could house these organisms, then the material itself would become a source of microbes. We would need to be very careful to select microorganisms that will not lead to serious health complications; although, all houses are already replete with human‐derived bacteria, fungi and viruses derived from the occupants. The majority of microbes from animals, plants or soil are unlikely to be overtly pathogenic, and so further selection among these would be relatively simple. Adding microbes from animals, plants, or soil, into this environment in a way that means that the animals, plants or soil themselves do not need to be present, would enable new buildings to be ‘probiotic’, or ‘healthy promoting’.

In addition, including microbes that can either out‐compete or directly inhibit the growth of less‐desirable organisms would provide additional benefits. For example, certain bacteria found in soil, as well as those in animals and plants, can inhibit the germination of fungi. Therefore, having walls impregnated with these bacteria could help to inhibit fungal growth during periods of water incursion. Mold and fungal growth after flooding in homes has serious health complications that require expensive cleanup or demolition of the structure. Water incursion into a material would activate spores or quiescent strains of anti‐fungal bacteria, inducing them to produce metabolites that would suppress fungal germination and thereby inhibit growth. The introduction of organisms that can out‐compete dangerous pathogens could also be beneficial. For example, methicillin‐resistant *Staphylococcus aureus* (MRSA) can be outcompeted in the human nares by *Staphylococcus lugdunensis* (Zipperer *et al*., 2016). Therefore, adding *S. lugdunensis* to building materials may help to reduce the spread of MRSA. However, there is a complication, in that this common skin‐associated bacteria has been associated with infections following cuts or skin injury, albeit ones that can be readily treated. However, it is possible that there are other bacteria or viruses that are more suitable, or that the metabolites produced by these organisms could be isolated and added to materials so that the biological organism need not be present.

The situation in hospitals in far more complex. Yet, it is still possible to imagine recovery rooms that have bioactive surface materials that could passively encourage healing in patients through immune stimulation. The alternative may be just to provide relevant probiotics through oral consumption or skin application as a cream. But, as some of the main routes of microbial acquisition and immune system activation are not fully understood, attempts to recreate a microbial exposure environment that provides a less direct association with the probiotic may prove to be beneficial. We are already exploring ways to augment the environment of microbes in the human colon during surgery to reduce nutrient stress and thereby reduce virulence activation. In animal studies, adding nutrients to the gut, that remain unavailable to the microbes, but allow them to sense the availability of nutrients, has been shown to substantial improve recovery rates (Zaborin *et al*., 2014). This paradigm of treating not only just the patient, but also the patients' microbiome, could become one of the changes in surgical practice over the next 5–10 years that has the biggest impact on beneficial outcomes.

Microbiologically inspired design and biotechnology has so many potential avenues for improving human health. And when it comes to altering our world to improve the health of our children, all options should be on the table. Determining the most effective way forward will require some very well designed experiments, and even a cultural shift in how we see microbes. Intervention studies that demonstrate health improvements from biologically active building materials will be the gold standard, but until then engineers, architects and policy makers need to collaborate with microbiologists and clinicians to identify the strategic way‐marker that need to be reached in order to determine the relevancy of such interventions. The paradigm of bringing the microbial world to the people, rather than the people to the microbial world, requires a Kuhnian shift that the public and policy makers may not be ready for, but that scientists and engineers needs to explore nevertheless.
